# An Investigation Into the Use of mHealth in Musculoskeletal Physiotherapy: Scoping Review

**DOI:** 10.2196/33609

**Published:** 2022-03-11

**Authors:** Jonathon M R Agnew, Catherine E Hanratty, Joseph G McVeigh, Chris Nugent, Daniel P Kerr

**Affiliations:** 1 Discipline in Physiotherapy School of Life and Health Sciences University of Ulster Newtownabbey United Kingdom; 2 Discipline of Physiotherapy School of Clinical Therapies, College of Medicine and Health University College Cork Cork Ireland; 3 Discipline in Computing School of Computing University of Ulster Newtownabbey United Kingdom

**Keywords:** physiotherapy, musculoskeletal, mHealth, rehabilitation, scoping review, mobile phone

## Abstract

**Background:**

Musculoskeletal physiotherapy provides conservative management for a range of conditions. Currently, there is a lack of engagement with exercise programs because of the lack of supervision and low self-efficacy. The use of mobile health (mHealth) interventions could be a possible solution to this problem, helping promote self-management at home. However, there is little evidence for musculoskeletal physiotherapy on the most effective forms of mHealth.

**Objective:**

The aim of this review is to investigate the literature focusing on the use of mHealth in musculoskeletal physiotherapy and summarize the evidence.

**Methods:**

A scoping review of 6 peer-reviewed databases was conducted in March 2021. No date limits were applied, and only articles written in the English language were selected. A reviewer screened all the articles, followed by 2 additional researchers screening a random sample before data extraction.

**Results:**

Of the 1393 studies, 28 (2.01%) were identified. Intervention characteristics comprised stretching and strengthening exercises, primarily for degenerative joint pain and spinal conditions (5/28, 18%). The most reported use of mHealth included telephone and videoconferencing calls to provide a home exercise program or being used as an adjunct to physiotherapy musculoskeletal assessment (14/28, 50%). Although patient satisfaction with mHealth was reported to be high, reasons for disengagement included a lack of high-quality information and poor internet speeds. Barriers to clinical uptake included insufficient training with the intervention and a lack of time to become familiar.

**Conclusions:**

mHealth has some benefits regarding treatment adherence and can potentially be as effective as normal physiotherapy care while being more cost-effective. The current use of mHealth is most effective when ongoing feedback from a health care professional is available.

## Introduction

### Background

Musculoskeletal conditions can have a major impact on people’s quality of life, leading them to seek medical care in the form of nonsteroidal anti-inflammatory drugs or surgery (eg, joint replacements), with people aged 55 to 65 years being the most common age group experiencing these conditions [1]. Musculoskeletal physiotherapy can provide cost-effective management for multiple conditions via modalities, including strengthening and flexibility exercises, postural and ergonomic advice, manual therapy (eg, joint mobilizations and soft tissue massage), and education for self-management of pain [2]. Effective physiotherapy helps improve short-term pain and disability, which facilitates earlier discharge from care [3], lowering the burden on the health care system by reducing waiting lists and financial costs [4]. Chronic conditions can result in pain and sickness-related absence from work and in patients seeking additional care up to 10 years after first receiving treatment, primarily for conditions with the highest recurrence rates such as low back and neck pain [5]. A possible contribution to the lack of success with treatment for chronic musculoskeletal issues is the lack of adherence to home exercise programs, low self-efficacy, failure to recall coping strategies, or lack of education provided by the therapist [6]. Furthermore, ongoing engagement with self-management is an important predictor of successful rehabilitation [7], and a series of focus groups of musculoskeletal physiotherapists have reinforced this regarding the management of patients with subacromial impingement syndrome [8]. A person-centered approach to treatment should be taken to encourage prolonged engagement with exercise [9]. Studies have concluded that patients prefer individualized, supervised exercise programs with clinician input [10,11]. An increasingly popular tool in a range of health care settings is the development of exercise programs delivered through mobile devices. An ideal app would enable web-based input from the clinician to support the patient to participate in rehabilitation from the comfort of their home [12]. There is evidence suggesting that the use of mobile apps with input from clinicians, particularly with the ability to set and monitor the quality of completion of the exercise, leads to higher adherence rates than traditional paper handouts [13].

eHealth is an umbrella term that refers to the use of modern information and communication technology to deliver health care [14]. A branch of eHealth showing growth in development is mobile health (mHealth) [15] as a result of the increasing use of mobile devices, partnered with improvements in technology development (eg, smartphones), with predictions that device availability will increase over the next decade [16]. According to the 2019 Ofcom report, the UK telecom sector generated £33.8 billion (US $45.03 billion), with mobile devices accounting for 51% of the total revenue. The average individual broadband data use increased from 30 GB per month in 2013 to 240 GB per month in 2018, whereas mobile data use increased by 37% from 2018, indicating increased access to internet-powered devices. This report also states that smartphones account for 60% to 90% of all telecommunications use for people aged 16 to 64 years, with those aged 16 to 34 years accounting for the largest proportion within this range. There is some evidence that younger patients may be more likely to engage in rehabilitation through the use of smartphones [17], although this does not mean that the older population is disadvantaged, as there is evidence showing that mHealth adherence is high throughout all age groups [18]. Other smart devices, including tablets and laptops, are mainly used by people aged 45 to 54 years, accounting for approximately 60% of smart device use, not including smartphones [19].

This innovative branch of health care has increased accessibility and affordability for patients [20], providing health care to patients with low income or those in rural locations where face-to-face health care is not practical [21]. There is already evidence of mHealth being implemented successfully to improve medication adherence [22]. Within health care settings, mental health and diabetes appear to have higher numbers of mHealth interventions with positive health outcomes [23-25]. Success in the management of mental health is because of the strict governance put in place by popular app sites such as Google Play and the App Store, alongside a larger research base behind these conditions [26]. The Developer Program Policies, along with the Developer Distribution Agreement [27], provide clear guidelines to developers. This ensures that any app being made widely available must be transparent with how it manages the user’s data, combined with ensuring that it contains appropriate content.

Another factor contributing to the rapid development of mHealth apps is the COVID-19 pandemic [28]. Owing to the need for whole populations to isolate, face-to-face appointments are being considered high risk, resulting in many patients still being in urgent need of treatment [29]. It has become vital to implement strategies that promote access to remote health care. The most viable and safe option has been to increase the number of mHealth apps being made available [30].

With the rise in smartphone availability, there has been a concomitant increase in research involving mobile device apps (mHealth) for the management of chronic pain [31]. The mHealth apps can be generalized into three main categories—(1) education, (2) pain measurement, and (3) pain therapy—with some apps falling into ≥1 category [32]. The third category potentially represents an intervention with the possibility of increasing the quality of life and function. Some mHealth apps require input from clinicians, whereas others do not. The latter presents fewer barriers, such as the user not needing to rely on an assessment from a clinician before use; however, a lack of clinician input may lead to disengagement and potentially risk an incorrect selection of exercises because of the lack of a working diagnosis [33]. This potentially represents a fourth category for mHealth, namely self-management. This, if applied effectively, gives the patient ownership of their own treatment—an important predictor of successful rehabilitation [34]. Despite this increase in research, there is still a need for specific research relating to musculoskeletal physiotherapy.

### Rationale

Little evidence underpins which aspects of mHealth are most effective and allow for the greatest level of engagement regarding musculoskeletal conditions [35]. A recent randomized controlled trial (n=68 participants) [2] compared an internet-based app supported by FitBit (Google LLC) with telephone-based health coaching sessions and an information booklet, with the advice to stay active by using the information booklet. Participants receiving the mHealth intervention had a 38% reduced rate of care seeking; however, statistical differences between groups were not reached regarding primary or secondary outcomes. Therefore, the authors could only state a possible advantage of using mHealth, with a more adequately powered trial needed. This trial relates to the current findings of research on mHealth in musculoskeletal physiotherapy, with a consensus on more rigorous research being needed, as the effectiveness of these interventions is not conclusive [36,37]. Research on mHealth within general physiotherapy has focused on treatment for respiratory conditions such as chronic obstructive pulmonary disease [38] or the views of therapists’ use of the interventions [39]. Previous systematic reviews conducted in this area of physiotherapy focused on multiple chronic diseases such as asthma, diabetes, and cancer [40,41]. Other systematic reviews that focused on physiotherapy mHealth interventions reported on diabetes mellitus and Duchenne muscular dystrophy, focusing on the features of the mHealth intervention compared with the clinical use of the intervention [42,43]. There is a gap in the research regarding the use of mHealth in musculoskeletal physiotherapy; therefore, there is scope for this review to be undertaken.

The aim of this review is to explore and chart the evidence on the use of mHealth within musculoskeletal physiotherapy, with a view to identifying relevant gaps in the literature by conducting a structured, systematic scoping review and developing relevant themes of the topic in question to address the feasibility of mHealth interventions.

## Methods

### Overview

This scoping review was conducted in accordance with a standardized framework [44]. This review was structured according to the five stages of this framework: (1) identifying the research question; (2) identifying relevant studies; (3) study selection; (4) charting the data; and (5) collecting, summarizing, and reporting the results. This scoping review was also guided by the PRISMA-ScR (Preferred Reporting Items for Systematic Reviews and Meta-Analyses extension for Scoping Reviews) [45].

### Stage 1: Identifying the Research Question

#### Objectives

The primary objective was to analyze the use of mHealth and the outcomes it had produced in musculoskeletal physiotherapy (eg, pain reduction and reported increase in self-efficacy). The secondary objectives were to determine the following: how mHealth has previously been applied, the types of conditions mHealth has been used for, interventions that have been proposed and implemented using mHealth, the reasons for barriers to and facilitators of mHealth, and the barriers to clinical uptake.

#### Eligibility Criteria

Studies were assessed against the inclusion and exclusion criteria described in Textbox 1.

**Inclusion and exclusion criteria for the studies.**

**Inclusion criteria**

- English language articles
- Peer-reviewed articles published in journals where full text was available
- Focus on the use and application of mobile health in musculoskeletal physiotherapy, including in patients and therapists
- Application of mobile health could be in an outpatient or home-based setting
- Studies in which mobile health was used as a whole or partial aspect of treatment combined with or without other modalities

**Exclusion criteria**

- Studies focusing on mobile health in other areas of health care (eg, as mental health and diabetes)

### Stage 2: Identifying Relevant Studies

Peer-reviewed articles were identified using key databases, including MEDLINE, Embase, ProQuest Health and Medical Complete, CINAHL Plus, AMED, and IEEE Xplore. These databases were chosen as they include a large collection of literature related to physiotherapy research alongside literature on health technology. Gray literature was also searched to allow for the inclusion of further relevant studies that were not identified through database searches. The search was conducted in March 2021.

The search strategy (Multimedia Appendix 1) used the terms *mHealth*, *eHealth*, or *Telemedicine* to identify articles related to the application of mHealth within physiotherapy. The reference lists of the appropriate articles were also snowball searched to identify any further literature.

The database searches were undertaken by three researchers (JMRA, DK, and CH) to identify all relevant literature, with no date limitations being applied to capture as much relevant literature as possible.

### Stage 3: Study Selection

All relevant references were imported into RefWorks (ProQuest), and duplicates were removed. One of the researchers (JMRA) applied the eligibility criteria for both the title and abstract review and full-text review stages. To allow for consensus on the eligibility criteria, 10% of the selected studies were reviewed by two additional researchers (DK and CH). This was followed by an assessment of the full texts of the included articles for the final inclusion stage by three researchers (JMRA, DK, and CH).

### Stage 4: Charting the Data

A data-charting form was developed to steer the collection of data from the included studies. This form included general data such as author and publication year, as well as more specific information relevant to this review. The data-charting form was piloted using a random selection from the database search results. This informed us of any changes needed before charting the data from the remaining studies. One of the researchers (JMRA) subsequently charted the data from all remaining studies, with 3 additional researchers reviewing a selection of these studies to ensure extra rigor.

### Stage 5: Collating, Summarizing, and Reporting the Results

A quantitative overview of the included studies was summarized in a series of tables and diagrams to aid in the synthesis of the literature related to the use of mHealth in physiotherapy. This included aspects such as which countries were applying mHealth, the nature of the intervention, and the common conditions for which mHealth was used. The final extracted data were also presented in a narrative account in the literature. The research team developed themes and categories that emerged with aid from both the research question and data produced using an iterative process.

## Results

### Study Selection

**Figure 1:**
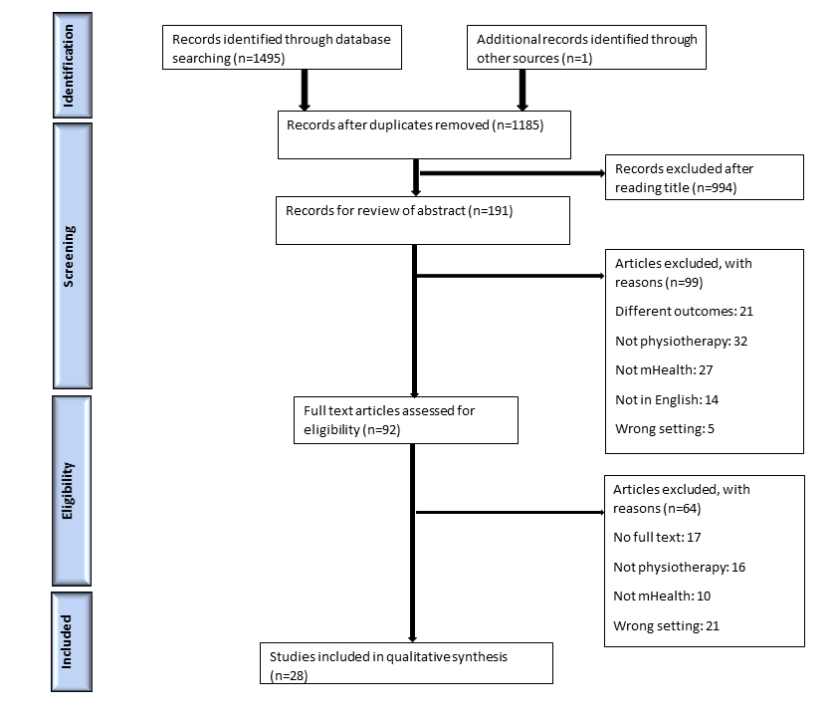
PRISMA (Preferred Reporting Items for Systematic Reviews and Meta-Analyses) flow diagram. mHealth: mobile health.

The initial database search (Multimedia Appendix 1) of the mHealth literature identified 1495 titles. Of these 1495 titles, 311 (20.8%) were duplicates. An additional 66.42% (993/1495) of studies were removed following title review as they did not meet the eligibility criteria. Of the 1495 titles, after an abstract review of 191 (12.78%) titles, 99 (51.8%) articles were removed; 21 (21%) articles were removed because of incorrect outcomes, 32 (32%) were removed because they did not focus on physiotherapy, 27 (27%) were removed because mHealth was not included, 14 (14%) were removed because they were non-English articles, and 5 (5%) were removed because they were studies conducted in settings not included in this review (ie, an inpatient hospital setting where mHealth may not be relevant as remote access would not be warranted). Of the 191 papers, the final full-text review of the remaining 92 (48.2%) papers provided 28 (14.7%) articles, with the reasons for exclusion involving no full-text availability in 17 (18%) papers, no focus on physiotherapy in 16 (17%) papers, mHealth not included in 10 (11%) papers, and 21 (23%) studies conducted in the incorrect setting as stated above. The search process is summarized in the flowchart (Figure 1).

### Study Characteristics

The study characteristics and findings are outlined in Tables 1-3. A total of 1393 participants were included in the final 28 included articles. The trial sample sizes ranged from 3 to 368 participants.

**Table 1:** Study characteristics.

Study	Study type	Location	Participants, N	Setting
Adamse et al [46]	Systematic review	The Netherlands	Not stated	Participants: aged >18 years Condition: chronic pain in any physical location Health care setting: —^a^
Adhikari et al [47]	Retrospective pre–post design	Nepal	15	Health care setting: rural home
Azma et al [48]	Randomized clinical trial	Iran	54	Participants aged 50 to 60 years Health care setting: home based or office based
Bini and Mahajan [49]	Randomized control study	United States	51	Health care setting: home based or face to face
Chen et al [50]	Pilot study to assess feasibility	Taiwan	15	Health care setting: home based
Correia et al [51]	Prospective parallel-group feasibility study	Portugal	69	Health care setting: home based
Dunphy et al [52]	Semistructured interviews	United Kingdom	24	Health care setting: outpatients
Eriksson et al [53]	Qualitative interviews	Sweden	10	Health care setting: home based
Eriksson et al [54]	Controlled study	Sweden	22	Health care setting: home based
Gialanella et al [55]	Prospective randomized controlled study	Italy	100	Health care setting: home based
Irvine et al [56]	Randomized controlled trial	United States	368	Health care setting: home based
Jay et al [57]	Randomized controlled trial	Denmark	38	Health care setting: office based
Lade et al [58]	Unclear	Australia	10	Health care setting: outpatients
Lawford et al [59]	Semistructured interviews	Australia	20	Health care setting: —^a^
Lovo et al [60]	Semistructured interviews analyzed using a mixed methods design	Canada	64	Health care setting: urban or home based
Mani et al [61]	Systematic review	Malaysia	—^a^	—^a^
Mecklenburg et al [62]	Randomized controlled trial	United States	162	Health care setting: home based
Meijer et al [63]	Systematic review	The Netherlands	—^a^	—^a^
Nelson et al [64]	Randomized controlled noninferiority trial	Australia	70	Health care setting: home based
Pastora-Bernal et al [65]	Single-blind prospective randomized clinical trial	Spain	18	Health care setting: home based
Peterson [66]	Case series	United States	3	Health care setting: home based
Piqueras et al [67]	Randomized controlled trial	Spain	142	Health care setting: outpatients or home based
Richardson et al [68]	Repeated measures design	Australia	18	Health care setting: outpatients
Rothgangel et al [69]	Prospective single-group clinical study	The Netherlands	15	Health care setting: private practice outpatients
Russell et al [70]	Repeated measures design	Australia	15	Health care setting: outpatients
Shukla et al [71]	Systematic review and meta-analysis	India	—^a^	—^a^
Tousignant et al [72]	Randomized controlled trial	Canada	48	Health care setting: home based
Wijnen et al [73]	Nonrandomized controlled trial combining a single-arm intervention cohort with historical controls	Netherlands	42	Health care setting: home based

^a^Not available.

**Table 2:** Study interventions and conditions.

Study	Condition	Intervention
Adamse et al [46]	Chronic pain to include chronic low back pain, osteoarthritis of the knee or hip, and rheumatoid arthritis	Telemedicine: internet-based technology used to communicate with patients to provide remote rehabilitation
Adhikari et al [47]	Prolapsed intervertebral disk, tennis elbow, rheumatoid arthritis, mechanical low back pain, traumatic ankle pain, and neck pain	Exercise pamphlets provided Via calls (4 times in 4 weeks); physiotherapist aided in the rehabilitation
Azma et al [48]	Knee osteoarthritis	Pamphlets provided (strengthening, endurance, flexibility, and ROM^a^ exercises) Continue exercises 3 times per week for 6 weeks Patients remotely contacted weekly regarding exercise progression
Bini and Mahajan [49]	Total knee replacement	CaptureProof app provided 23 exercise videos Videos narrated by a therapist with on-screen instructions Patient responds with a recording of their exercise completion Therapist reviews and adjusts treatment as appropriate
Chen et al [50]	Shoulder adhesive capsulitis	MSD^b^ measures ROM Patient app used by patient and physician app used by a health care professional Effectiveness of rehab measured using patient and physician app
Correia et al [51]	Total knee arthroplasty	Physiotherapist trained patient or caregiver in the use of the platform Sessions performed 5 times per week for a minimum of 30 minutes
Dunphy et al [52]	ACL^c^ reconstruction	Interviews with physiotherapists and patients
Eriksson et al [53]	Shoulder joint replacement	Patients supervised by a physiotherapist Physiotherapist contacted patient via videoconferencing
Eriksson et al [54]	Shoulder joint replacement	Patients supervised by a physiotherapist Physiotherapist contacted patient via videoconferencing
Gialanella et al [55]	Chronic neck pain	HBT^d^ group comprising fortnightly calls Unscheduled calls in the event of uncontrolled pain Advice on exercise, disease status, pain, and disability provided
Irvine et al [56]	Sedentary behavior in older adults	Active after 55 to 12 sessions, 10 to 15 minutes each More challenging exercises progressively introduced SMS text messages and video messages to assist with goal setting
Jay et al [57]	Upper limb musculoskeletal pain	Video-based exercises showing correct performing of exercises Audio instructions provided for each exercise Web-based instructional material also made accessible
Lade et al [58]	Musculoskeletal elbow disorders	Participants were interviewed and examined face to face and remotely via a telerehabilitation system
Lawford et al [59]	Knee osteoarthritis	Participants received 5 to 10 telephone calls over 6 months Initial calls lasted approximately 40 minutes, with follow-up calls lasting 20 minutes Action plan involving home strengthening exercise program and physical activity plan were devised Program and goals adjusted as necessary
Lovo et al [60]	Chronic back disorder management	Urban PT^e^ joined with NP^f^ via telehealth to undergo a full neuromusculoskeletal lumbar spine assessment Patients provided with a summary of findings and answers to questions
Mani et al [61]	Musculoskeletal disorders assessments	Validity and inter- and intrarater reliabilities of telerehabilitation-based physiotherapy examined Two independent reviewers used QAREL^g^ and QUADAS^h^ to assess the methodological quality
Mecklenburg et al [62]	Chronic knee pain	Hinge health delivered remotely for 12 weeks Information provided for exercise therapy, education, CBT^i^, weight loss, and psychosocial support
Meijer et al [63]	Traumatic bone and soft tissue injuries	A total of 12 articles were included No studies on wearable-controlled games or rehabilitation games included All studies were low to moderate quality
Nelson et al [64]	Total hip replacement	Remotely delivered telerehabilitation into the home Technology-based HEP^j^ provided using iPad app
Pastora-Bernal et al [65]	Subacromial decompression	Customized exercises through a web application Participants received 12-week (5 days per week) video exercises alongside a telerehabilitation patient manual
Peterson [66]	Chronic low back pain	Participants tracked daily pain levels and HEP adherence using a mobile phone app for 12 months following discharge
Piqueras et al [67]	Total knee arthroplasty	IVT^k^ comprising 1-hour sessions for 10 days (5 performed under supervision and 5 performed at home)
Richardson et al [68]	Musculoskeletal disorders of the knee	Patient interview and face-to-face and web-based assessment via telerehabilitation system Telerehabilitation assessments involved facilitated self-palpation, self-applied modified orthopedic tests, and active movements and functional tasks
Rothgangel et al [69]	ACL reconstruction	A total of 7 Dutch private practices participated in this study Data collected regarding physiotherapists’ most used components, acceptability, and suggested improvements
Russell et al [70]	Musculoskeletal ankle disorders	Patient interviews conducted face to face and on the web via telerehabilitation Web-based assessment recorded via eHAB system to allow for interrater and intrarater reliability components to be performed
Shukla et al [71]	Total knee arthroplasty	Six publications included Patients experienced high levels of satisfaction with telerehabilitation alone No changes to outcomes of active knee extension and flexion
Tousignant et al [72]	Total knee arthroplasty	16 telerehabilitation sessions over 2 months Conducted via videoconferencing delivered to patients’ home
Wijnen et al [73]	Total hip arthroplasty	12-week home-based telerehabilitation program with instructions provided via a web-based app Strengthening and walking exercises of the affected hip included Remote coaching provided via weekly telephone calls Recommendations were given regarding exercise progression

^a^ROM: range of motion.

^b^MSD: motion sensor device.

^c^ACL: anterior cruciate ligament.

^d^HBT: home-based telemedicine.

^e^PT: physical therapist.

^f^NP: nurse practitioner.

^g^QAREL: Quality Appraisal tool for studies of diagnostic reliability.

^h^QUADAS: Quality Assessment of Diagnostic Accuracy Studies.

^i^CBT: cognitive behavioral therapy.

^j^HEP: home exercise program.

^k^IVT: interactive virtual telerehabilitation.

**Table 3:** Outcome measures and findings.

Study	Outcome measures	Findings
Adamse et al [46]	Outcome measure not stated	Telemedicine vs no intervention showed lower scores for pain (MD^a^ –0.57, 95% CI –0.81 to –0.34) Nonsignificant effects shown for function (MD 19.93, 95% CI –5.20 to 45.06 minutes per week)
Adhikari et al [47]	Pain: NPRS^b^	NPRS demonstrated significantly decreased pain: at rest: F=3.5, P<.04; when worst: F=26.4, P<.001; during activity: F=16.6, P<.001; during occupation: F=15.6, P<.001
Azma et al [48]	Pain: KOOS^c^ Function: WOMAC^d^	In both groups, KOOS scores increased from baseline to 6 months (50.6 to 83.1 and 49.8 to 81.8) No significant difference in either group in any of the studied scales
Bini and Mahajan [49]	PRO^e^: VAS^f^, VR-12^g^, and KOOS-PS^h^	No statistically significant difference between groups on any outcome Overall use of hospital resources 60% less than traditional group
Chen et al [50]	Pain: VAS Function: qDASH^i^ Exercise completion rate: self-reported and motion sensor data	MSD^j^ exhibited good to excellent reliability for shoulder ROM^k^ (intraclass correlation coefficient range 0.771-0.979) MSD rehab assisted group displayed better shoulder mobility and function
Correia et al [51]	Primary outcomes: TUG^l^ score Secondary outcomes: KOOS and knee ROM in degrees	For primary outcome at 6 months, the median difference between groups was 4.87 (95% CI 1.85 to 7.47) seconds in favor of the intervention group
Dunphy et al [52]	Interviews analyzed using pragmatic thematic analysis	Patients’ six themes: experience of TRAK^m^, reasons for engagement, strengths, weaknesses, future use, and attitudes to digital health care Physiotherapists’ three themes: potential benefits, availability of resources, and service organization to support TRAK
Eriksson et al [53]	Qualitative content analysis	Six categories were identified: a different reinforced communication, pain-free exercising as an effective routine, from a dependent patient to a strengthened person at home, closeness at a distance, facilitated daily living, and continuous physiotherapy chain
Eriksson et al [54]	Pain: VAS Function: Constant-Murley ROM: Goniometer Shoulder condition: SRQ-S^n^	Statistically significant improvements in all outcomes for both groups, with the telemedicine group improving more (P<.001 for all)
Gialanella et al [55]	Pain: VAS Function: Neck Disability Index	At 6 months, neck pain and disability decreased in both groups (P<.001), with the decline being more marked in HBT^o^ group (P=.001) 87.2% of patients undergoing HBT and 65.9% of control participants were performing home exercises (2-7 sessions per week)
Irvine et al [56]	Self-reported 14-point questionnaire measuring physical activity status to behavioral intentions to change	At posttest, intervention participation showed significant improvement on 13 of 14 outcome measures compared with control participants At 6 months, intervention participants maintained large improvements on all 14 outcomes compared with control participants
Jay et al [57]	Descriptive statistics: training frequency, use of written and video material, training adherence, and pre- to posttraining self-perceived pain of the neck, shoulder, arm, and wrist	Unilateral shoulder external rotation had a higher normalized error score in the V group of 22.19 (SD 9.30) to 12.64 (SD 6.94) in the *P* group (P=.002)
Lade et al [58]	Unclear	There was substantial agreement for validity in systems diagnosis (73%; P=.01) Almost perfect intrarater reliability (90%; P=.001) Interrater reliability had a weaker agreement (64%; P=.11)
Lawford et al [59]	Thematic analysis	Participants described positive experiences with received therapy via telephone, valuing convenience and accessibility Some desired visual contact with the physiotherapist Participants valued undivided attention from the physiotherapist and were able to communicate effectively over the phone Participants felt confident performing their exercise program without supervision
Lovo et al [60]	Interviews analyzed qualitatively and quantitatively	Patients were very satisfied (62.1%) or satisfied (31.6%) with the overall experience Patients were very (63.1%) or somewhat (36.9%) confident with the assessment
Mani et al [61]	Methodological quality: QAREL^p^ and QUADAS^q^	11 articles were reviewed Studies were moderate to good in quality Physiotherapy assessments of pain, swelling, ROM, muscle strength, balance, gait, and functional assessment demonstrated good validity Low to moderate validity for lumbar spine posture, special orthopedic tests, neurodynamic tests, and scar assessments
Mecklenburg et al [62]	Pain: KOOS Function: KOOS-PS	Digital care program demonstrated a statistically significantly higher reduction in pain (7.7, 95% CI 3.0 to 12.3; P=.002) A statistically significantly greater improvement in function (7.2, 95% CI 3.0 to 11.5; P=.001)
Meijer et al [63]	Outcome measures not stated	12 studies were included Studies were low to moderate quality 2 studies found beneficial effects of serious games compared with conventional therapy 1 of 3 studies found beneficial effects of serious games 1 of 5 trials found a statistically significant advantage in the serious game group regarding treatment adherence
Nelson et al [64]	Function: SF-12^r^ QoL^s^: HOOS^t^ subscale	No between-group difference detected in the HOOS subscale (P=.97) Strength, balance, and self-reported function showed no between-group difference
Pastora-Bernal et al [65]	Function: Constant-Murley	Telerehabilitation group was shown to have improved functional outcome: mean of 43.5 (SD 3.21) points and 68.5 (SD 0.86) points after 12 weeks
Peterson [66]	Function: Oswestry Disability Index	All patients met their individual goals Excellent home exercise program adherence was displayed Temporary increase in pain was noted; however, patients managed via telerehabilitation booster sessions and no other resources
Piqueras et al [67]	Function: WOMAC Muscle strength, walk speed, and pain data collected	All participants improved after the 2-week intervention on all outcomes (P<.05) Telerehabilitation group achieved similar functional improvements to the control group
Richardson et al [68]	Reference given to assessment findings measured via Likert and binary scales	System of pathology in agreement in 17 (94%) out of 18 cases Comparisons of objective findings demonstrated substantial agreement (Cohen *κ*=0.635) for categorical and binary data (*χ^2^*=400.4; P<.001) High intrarater (89%) and moderate interrater (67%) reliability was evident for telerehabilitation assessments
Rothgangel et al [69]	Data regarding platform use and acceptance measured using 7- and 11-point numerical scales	Platform use was generally limited, with the number of log-ins ranging from 3 to 73 Overall, therapists’ acceptance was low to moderate Average scores ranged from 2.5 (SD 1.1) to 4.9 (SD 1.5)
Russell et al [70]	Clinical observations rated on a series of Likert and binary scales	Similar agreement (93.3%) was found in pathoanatomical diagnoses An 80% agreement (*χ^2^*=4.3; P<.04) in primary systems diagnoses found between face-to-face and web-based assessments Very strong agreement (*κ*=.92) for categorical data and significant agreement (93.3% agreement; *χ^2^*=234.4; P<.001) for binary data
Shukla et al [71]	Pain: VAS Functional assessment: TUG test Functional capacity: WOMAC Knee movement and quadriceps strength	Six studies included No statistically significant difference in change in active knee extension or flexion in the home telerehabilitation group compared with the control group (MD −0.52, 95% CI −1.39 to 0.35, P=.24 and MD 1.14, 95% CI −0.61 to 2.89, P=.20)
Tousignant et al [72]	Function: WOMAC QoL: SF-36^u^ Disability: 30-second chair stand test	Clinical outcomes improved significantly in both groups between end points Some variables showed larger improvements in the usual care group 2 months after discharge
Wijnen et al [73]	Function: TUG test, HOOS, five times Sit-to-Stand test QoL: SF-36	Intervention group performed functional tests significantly faster at 12 weeks and 6 months postoperatively Large effect sizes were found on functional tests at 12 weeks and 6 months (Cohen d=0.5-1.2)

^a^MD: mean difference.

^b^NPRS: Numerical Pain Rating Scale.

^c^KOOS: Knee Osteoarthritis Outcome Score.

^d^WOMAC: Western Ontario and McMaster Universities Osteoarthritis Index.

^e^PRO: patient-reported outcome.

^f^VAS: visual analog scale.

^g^VR-12: Veterans-RAND 12.

^h^KOOS-PS: KOOS short form.

^i^qDASH: Quick Disabilities of the Arm, Shoulder, and Hand.

^j^MSD: motion sensor device.

^k^ROM: range of motion.

^l^TUG: Timed Up and Go test.

^m^TRAK: Taxonomy for RehAbilitation of Knee conditions.

^n^SRQ-S: Shoulder Rating Questionnaire.

^o^HBT: home-based telemedicine.

^p^QAREL: Quality Appraisal tool for studies of diagnostic reliability.

^q^QUADAS: Quality Assessment of Diagnostic Accuracy Studies.

^r^SF-12: 12-Item Short Form Health Survey.

^s^QoL: quality of life.

^t^HOOS: Hip disability and Osteoarthritis Outcome Score.

^u^SF-36: 36-Item Short Form Health Survey.

### Study Design

Overall, there were more quantitative studies (23/28, 82%) than qualitative studies (4/28, 14%; Table 2). There were only 4% (1/28) of mixed methods studies. The most common study type was randomized controlled trials (10/28, 36%), followed by systematic reviews (4/28, 14%), one of which included a meta-analysis. The various forms of randomized controlled trials included randomized controlled trials (7/28, 25%), prospective randomized controlled trials (2/28, 7%), and randomized controlled noninferiority trials (1/28, 4%). Other quantitative designs included repeated measures design (2/28, 7%), retrospective pre–post design (1/28, 4%), pilot study to assess feasibility (1/28, 4%), prospective parallel-group feasibility study (1/28, 4%), controlled study (1/28, 4%), prospective single-group clinical study (1/28, 4%), case series (1/28, 4%), and nonrandomized controlled trial combining a single-arm intervention cohort with historical controls (1/28, 4%). Qualitative designs included semistructured interviews (3/28, 11%). Only 4% (1/28) of studies were referred to only as a qualitative interview [53]. Mixed methods designs included 4% (1/28) of studies in which data were analyzed using a mixed methods design [60]. The remaining study design (1/28, 4%) was inadequately described [58].

### Study Location

A total of 15 geographical locations were reported in all the studies. These studies covered the continents of North America (6/28, 21%), Europe (12/28, 43%), Asia (5/28, 18%), and Oceania (5/28, 18%). The North American locations were divided into Canada (2/28, 7%) and the United States (4/28, 14%). The continent of Europe included the largest number of locations, including the Netherlands (4/28, 14%), Sweden (2/28, 7%), Spain (2/28, 7%), Portugal (1/28, 4%), the United Kingdom (1/28, 4%), Italy (1/28, 4%), and Denmark (1/28, 4%). Asia contained the next most locations, comprising Nepal (1/28, 4%), Iran (1/28, 4%), Taiwan (1/28, 4%), Malaysia (1/28, 4%), and India (1/28, 4%). Oceania included only Australia (5/28, 18%).

### Intervention Characteristics

Despite all studies stating mHealth as part of the intervention, a significant number of studies failed to adequately describe the input of mHealth to the extent that it would be reproducible. Several studies reported the intervention as being *an exercise program delivered to the patient’s home*; however, the exact nature of these protocols was not described in sufficient detail. Those studies that provided enough detail described the elements of strengthening [48,56,57,59,73] and stretching [48,56]. One of the studies described walking exercises [73], whereas another study included education, cognitive behavioral therapy, weight loss, and psychosocial support as part of the intervention [62]. Other studies explored the use of mHealth as an adjunct to physiotherapy assessment [58,60,68,70] to assess the inter- and intrareliability of remote assessments using telerehabilitation technologies.

### Findings

#### How mHealth Has Previously Been Applied

##### Previous Applications of Rehabilitative mHealth

Of the 28 included studies, 4 (28%) systematic reviews [46,61,63,71] and 1 (4%) other study [58] explored the previous applications of mHealth. Relevant studies within the systematic reviews were included separately in this review. The remaining studies focused on the feasibility and efficacy of current and future applications. Reports of previous applications of mHealth largely included telephone-based interventions using videoconferencing connected via the internet to the patients’ homes (4/28, 14%). Another study described the inclusion of an interactive web-based telerehabilitation software alongside videoconferencing, including wireless sensors to record patients’ movements, an interactive software to demonstrate the strengthening and range of motion (ROM) exercises undertaken following total knee arthroplasty, and a web portal for clinician input [71]. Other methods described in less detail referred to mHealth delivery via smartphones or the internet [46]. This study [46] also referenced that all interventions conducted in a home-based setting included an individually tailored exercise program alongside the promotion of self-management strategies such as chat sessions and group exercises. Other forms of mHealth applications included the use of rehabilitation games widely available on multiple platforms such as the Wii, PlayStation EyeToy, and Xbox Kinect to aid in rehabilitation following traumatic bone and soft tissue injuries. Many of these games involved balance and mobility exercises using Wii [63].

##### Previous mHealth Applications for Professional Use

Only 7% (2/28) of the studies [58,61] described the use of mHealth as an aid to the physiotherapy assessment of musculoskeletal disorders. The aim of these studies was to explore the validity of web-based assessment compared with traditional face-to-face methods. The inclusion of mHealth once again involved videoconferencing, in which the patient was required to self-palpate and perform modified self-administered special tests. The results showed that mHealth could be a valid alternative to accurately measuring several objective measures such as pain, ROM, muscle strength, gait, and swelling. However, the evidence was not strong enough to suggest that mHealth is a viable solution for measuring neurodynamic tests and spinal posture.

#### Types of Musculoskeletal Conditions Where mHealth Has Been Used

Although studies have reported the type of musculoskeletal condition for which mHealth was being used, some studies described a broader term covering a range of conditions within the same area (EG, musculoskeletal ankle disorders, musculoskeletal disorders of the knee, and sedentary behavior in older adults; Table 2). Among the adequately described musculoskeletal conditions, total knee replacement or arthroplasty (4/28, 14%) was the most common. Other surgical procedures where mHealth was used also included total hip replacement or arthroplasty (2/28, 7%), anterior cruciate ligament reconstruction (2/28, 7%), shoulder joint replacement (2/28, 7%), and subacromial decompression (1/28, 4%). Several articles explored chronic conditions such as chronic knee pain or knee osteoarthritis (3/28, 11%), chronic hip pain and hip osteoarthritis (1/28, 4%), shoulder adhesive capsulitis (1/28, 4%), chronic or mechanical low back pain (4/28, 14%), chronic neck pain (2/28, 7%), and rheumatoid arthritis (2/28, 7%). Less common conditions included prolapsed intervertebral disk (1/28, 4%) and tennis elbow (1/28, 4%).

#### Interventions That Have Been Implemented Using mHealth

There appears to be no novel intervention being implemented when compared with how mHealth has previously been applied. The main theme throughout most studies was the aspect of communication between the treating therapist and the patient to allow for a successful course of treatment involving mHealth. This could involve telephone calls (teleconferencing) or videoconferencing (eg, Skype [Microsoft Corporation]). The current articles suggest mHealth is best implemented as an adjunct to *usual care*, which can be defined as face-to-face physiotherapy involving exercise therapy and manual therapy [64]. A number of studies included pamphlets with the addition of weekly teleconferencing calls from participating clinicians [47,59]. For studies that did not include teleconferencing as a part of the intervention, a series of smartphone-based apps [49,66] and web-based applications were implemented [47,51,56,57,62,73]. These interventions included narrated videos of exercises with which the patient would respond by sending back recordings of them completing the exercise. This would allow for appropriate exercise progression via clinician inputs. One of the studies [65] involved the use of a wearable motion sensor device alongside an app for patients (patient app) and an app for clinicians (physician app). The patient app helped participants visualize the correct ROM of the exercises, and the physician app provided clinicians with a data log of participants’ progression, allowing for input via text.

#### Reasons for Engagement or Disengagement With mHealth

Approximately 64% (18/28) of articles stated the reasons for engagement or disengagement from the intervention. Overall, these reasons were not described in sufficient detail. In general, patient satisfaction was very high as participants valued the interactive features and readily available support as very important. Studies involving preoperative protocols reported that interest in surgery decreased as knowledge of their condition increased because of the constant engagement with their clinician [62]. It was also shown in several articles that mHealth increased long-term (defined as 6 months) adherence to treatment, as the influence of specialist supervision was shown to help maintain motivation and confidence in the process as well as constant goal setting [48,50]. Reasons for disengagement were stated as technological problems such as the speed of the internet connection and the *clunky* design of some of the apps [46,55]. However, it was stated that this could be minimized by implementing a web-based platform on mobile devices that could be used with standard data speeds, as most participants would be in possession of mobile devices capable of doing so [49]. It was reported that video-based interventions gave participants the most effective treatment as the videos informed them of the correct technique and gave them the confidence to perform the exercises correctly [55].

#### Barriers to mHealth Clinical Uptake

Only 4% (1/28) of the studies specifically explored the experience of clinicians in using mHealth [52]. This study reported the limited use of a novel telemonitoring device with a low to moderate acceptance rate among physiotherapists. A possible explanation for this was the lack of time to become familiar with the telemonitoring platform. The main issue among physiotherapists was the added workload that the intervention imposed, as therapists had to input data into an additional eHealth data log. Suggestions for future use included improvements in user-friendliness, efficiency, and design. Some therapists proposed integrating digital health technology into routine care to more easily become a new habit of clinical practice. A preference for smartphone-based apps over web-based applications was also reported, with no reasons adequately described. The final barrier suggested in this study was the lack of structured training given to current and future health care professionals to promote knowledge of new health care technologies. In the future, novel health care technologies should be more easily integrated into clinicians’ routines, and training should be provided alongside this.

## Discussion

### Principal Findings

This study represents a mapping of the breadth of evidence for the use of mHealth within musculoskeletal physiotherapy and identifies 5 themes of mHealth implementation, including facilitators of and barriers to uptake. The main aim of this scoping review was to analyze the evidence surrounding the use of mHealth in musculoskeletal physiotherapy and the outcomes it produced. The main findings from this review suggest that videoconferencing or phone calls are the most popular among patients as they provide ongoing feedback with a clinician, potentially leading to a higher adherence rate to rehabilitation programs. Another finding has shown a lack of adequate training in mHealth use among clinicians, leading to poor uptake.

This review demonstrates that there is potential in the future for mHealth to be a viable component of musculoskeletal physiotherapy care. Recent studies have proposed that mHealth interventions have the potential to be more effective than usual physiotherapy care, as the increased use of smartphones enables patients to source information and take control of their rehabilitation [69]. However, this review has shown limited evidence to support this claim, as only 11% (3/28) of studies [49,50,66] included the use of smartphones and only 4% (1/28) of studies compared mHealth with physiotherapy, concluding that a comprehensive digital care intervention, combined with ongoing support provided with normal physiotherapy care, significantly improves outcomes for pain and function [62]. The remainder of the studies either claimed that mHealth could potentially be at least as effective as physiotherapy or were inadequately described to make any conclusions.

There is limited evidence suggesting that mHealth can be effectively used for physiotherapy musculoskeletal assessments as an alternative to face-to-face assessments. Of the 28 studies, 2 (7%) studies [58,61] suggested that this form of assessment was both valid and reliable, with 1 (4%) investigating the specific assessment of the elbow [58] and 1 (4%) investigating general musculoskeletal disorders [61]. However, the evidence suggests that this is not an acceptable alternative as special neurodynamic tests were unable to be sufficiently conducted as the patient was unable to apply the tests as a clinician would, leading to unreliable findings. Telephone or videoconferencing calls between the therapist and patient were the most accepted forms of mHealth in musculoskeletal physiotherapy. This could be viewed as a potential pitfall unless further innovation is made in this field, as patients are more likely to respond positively to a readily available app on their smartphone [74]. Most research in other medical fields has concluded that telephone or videoconferencing calls are the most popular intervention, further emphasizing the need for more development [75,76]. It is important that development continues, as reports suggest that patients feel there is a lack of currently available, relevant high-quality mHealth apps providing adequate support [77].

A range of conditions was analyzed in this review, suggesting a lack of research on mHealth use for particular musculoskeletal conditions. Postoperative rehabilitation after total knee replacement was the most researched condition for mHealth use. Only 11% (3/28) of studies investigated mHealth for the treatment of chronic low back pain [46,60,66], and 14% (4/28) of studies were related to shoulder pain [51,53,65,78]. Therefore, there is little evidence to fully support the use of mHealth for a multitude of conditions.

Very few studies described the mHealth intervention in detail in a way that would be reproducible. As this review was conducted in the context of musculoskeletal physiotherapy, it can be assumed that physiotherapy care would be within the context of the intervention. Most authors failed to describe the physiotherapy component in detail, simply describing the intervention as *an exercise program delivered to the home, with follow-up telephone calls from a participating clinician*, with the assumption that this is a form of treatment rather than an umbrella term encompassing a range of interventions. This suggests that there is insufficient evidence to guide physiotherapists on how to effectively deliver an mHealth intervention, as supported by 4% (1/28) of the studies in this review [52].

This review highlights a lack of qualitative research on mHealth interventions as most evidence was quantitative in nature. The importance of understanding the experiences of those delivering and receiving these interventions is not to be understated and can be a vital part of enhancing the delivery of future interventions [54]. This can provide useful insights from both clinicians and patients on how to continually innovate mHealth and increase engagement and better patient care, as the value of qualitative research provides a richer insight into the lived experience [79]. This review has shown that the continents of North America, Asia, Europe, and Oceania currently have the strongest research output in support of the development of future mHealth interventions. It can be concluded that mHealth interventions are being implemented in high-income countries because of access to high-quality resources, infrastructure, and time to develop more effective and engaging interventions, including aspects such as gamification [80].

### Study Limitations

Although most evidence within this review was conducted within the past 10 years, we excluded articles that were non-English articles, implying the possibility of excluding relevant articles from non–English-speaking countries (eg, China, Japan, and South Korea), where technology is well-advanced [81]. In addition, a consultation stage was not included in the review process through which we may have gained more insight, and study authors were not contacted for additional information. When compared with systematic reviews, the absence of a strong quality assessment of papers in scoping reviews makes any findings difficult to generalize and presents challenges in weighting the effectiveness of studies [44]. Despite this, we believe that the breadth of the evidence presented is sufficient for the aims of this review.

### Research Opportunities and Recommendations

With the onset of the COVID-19 pandemic, alternatives to face-to-face musculoskeletal physiotherapy have become a priority. Future smart device–based mHealth interventions should focus on implementing evidence-based strategies in research design and using more innovative health care technologies to help enhance and expand the practice of mHealth. To aid in the development of the rapidly expanding market of mHealth, future research should look to develop evidence-based rehabilitation programs for acute and chronic conditions using the latest technologies and provide adequate training for clinicians.

### Conclusions

It appears that mHealth has some beneficial effects on treatment adherence and can be as effective as the usual physiotherapy care and potentially more cost-effective. Currently, communication with a clinician via telephone or videoconferencing appears to be the most widely accepted among patients, as this helps maintain confidence in their rehabilitation because of ongoing feedback. This feedback loop between the clinician and the patient potentially leads to positive outcomes regarding pain and self-management because of increased adherence to the rehabilitation program.

The limitations identified in this review provide an outline for future studies. This review has shown the main limitations to mHealth uptake from clinicians, primarily as a lack of knowledge and confidence in their judgment when using mHealth interventions and a preference toward an evidence-based clinical technique [57]. Researchers have suggested more widely available training for clinicians implementing mHealth interventions in the future. The barriers to uptake among patients are related to the user-friendliness and aesthetics of the intervention, as it is likely that patients will discontinue use after a short period because of the lack of an efficient design [82]. What constitutes an efficient mHealth design is not adequately described within this review, with the only exception suggesting the use of videos within an app to promote engagement; therefore, we propose further research with a focus on designing an implementation framework and designing trials investigating long-term adherence and the effect of clinicians trained in mHealth implementation on long-term treatment outcomes.

## Appendix

Multimedia Appendix 1 Search Strategy.

## Abbreviations

mHealth: mobile health
PRISMA-ScR: Preferred Reporting Items for Systematic Reviews and Meta-Analyses extension for Scoping Reviews
ROM: range of motion
