# Supplementary material for: An Investigation Into the Use of mHealth in Musculoskeletal Physiotherapy: Scoping Review
Source: JMIR Rehabil Assist Technol. 2022 Mar 11;9(1):e33609. doi: 10.2196/33609 (PMC8956993; doi:10.2196/33609)
Supplement: Multimedia Appendix 1 [file rehab_v9i1e33609_app1.pdf]

Search Strategy

Table	Search strategy
1.	exp Telemedicine/
2.	(mhealth or ehealth or m-health or e-health or mobile health or mobile healthcare or electronic health or electronic healthcare or telehealth or tele-health or telehealthcare or telemanagement or tele-management or interactive health communication or consumer health informatic* or health technolog* or connected health).mp. [mp=title, abstract, original title, name of substance word, subject heading word, floating sub-heading word, keyword heading word, organism supplementary concept word, protocol supplementary concept word, rare disease supplementary concept word, unique identifier, synonyms]
3.	1 or 2
4.	exp Physical Therapy Modalities/
5.	(physiotherap* or physical therap*).mp. [mp=title, abstract, original title, name of substance word, subject heading word, floating sub-heading word, keyword heading word, organism supplementary concept word, protocol supplementary concept word, rare disease supplementary concept word, unique identifier, synonyms]
6.	4 or 5
7.	exp Rehabilitation/
8.	(rehabilitat* or recover*).mp. [mp=title, abstract, original title, name of substance word, subject heading word, floating sub-heading word, keyword heading word, organism supplementary concept word, protocol supplementary concept word, rare disease supplementary concept word, unique identifier, synonyms]
9.	7 or 8
10.	exp Musculoskeletal/
11.	(musculoskeletal or msk).mp. [mp=title, abstract, original title, name of substance word, subject heading word, floating sub-heading word, keyword heading word, organism supplementary concept word, protocol supplementary concept word, rare disease supplementary concept word, unique identifier, synonyms]
12.	10 or 11
13.	6 and 9 and 12
14.	3 and 13
